# Flow diverter effect of LVIS stent on cerebral aneurysm hemodynamics: a comparison with Enterprise stents and the Pipeline device

**DOI:** 10.1186/s12967-016-0959-9

**Published:** 2016-07-02

**Authors:** Chao Wang, Zhongbin Tian, Jian Liu, Linkai Jing, Nikhil Paliwal, Shengzhang Wang, Ying Zhang, Jianping Xiang, Adnan H. Siddiqui, Hui Meng, Xinjian Yang

**Affiliations:** Department of Interventional Neuroradiology, Beijing Neurosurgical Institute and Beijing Tian Tan Hospital, Capital Medical University, TiantanXili 6, Dongcheng District, Beijing, China; Department of Neurosurgery, The Affiliated Hospital, Binzhou Medical University, Binzhou, Shandong China; Toshiba Stroke and Vascular Research Center, University at Buffalo, The State University of New York, Buffalo, NY USA; Department of Mechanical and Aerospace Engineering, University at Buffalo, The State University of New York, Buffalo, NY USA; Department of Mechanics and Engineering Science, Fudan University, Shanghai, China; Department of Neurosurgery, University at Buffalo, The State University of New York, Buffalo, NY USA

**Keywords:** Computational fluid dynamics (CFD), Wall shear stress (WSS), Intracranial aneurysm, LVIS, Hemodynamics

## Abstract

**Background:**

The aim of this study was to quantify the effect of the new Low-profile Visualized Intraluminal Support (LVIS®D) device and the difference of fluid diverting effect compared with the Pipeline device and the Enterprise stent using computational fluid dynamics (CFD).

**Methods:**

In this research, we simulated three aneurysms constructed from 3D digital subtraction angiography (DSA). The Enterprise, LVIS and the Pipeline device were virtually conformed to fit into the vessel lumen and placed across the aneurysm orifice. Computational fluid dynamics analysis was performed to compare the hemodynamic differences such as WSS, Velocity and Pressure among these stents.

**Results:**

Control referred to the unstented model, the percentage of hemodynamic changes were all compared to Control. A single LVIS stent caused more wall shear stress reduction than double Enterprise stents (39.96 *vs.* 30.51 %) and velocity (23.13 *vs.* 18.64 %). Significant reduction in wall shear stress (63.88 %) and velocity (46.05 %) was observed in the double-LVIS stents. A single Pipeline showed less reduction in WSS (51.08 %) and velocity (37.87 %) compared with double-LVIS stent. The double-Pipeline stents resulted in the most reduction in WSS (72.37 %) and velocity (54.26 %). Moreover, the pressure increased with minuscule extent after stenting, compared with the unstented model.

**Conclusions:**

This is the first study analyzing flow modifications associated with LVIS stents. We found that the LVIS stent has certain hemodynamic effects on cerebral aneurysms: a single LVIS stent caused more flow reductions than the double-Enterprise stent but less than a Pipeline device. Nevertheless, the double-LVIS stent resulted in a better flow diverting effect than a Pipeline device.

## Background

Endovascular stent-assisted coil embolization has been widely used for treatment of intracranial aneurysms. However, wide-necked, complex or dissecting aneurysms that incorporate a large portion of the parent artery can still be a challenge to treat and usually require scaffolding or bridging of the neck by special stents, combined with aneurysm coiling or flow diverters [1, 2]. The coil embolization refers to the filling of the aneurysm with coils and intracranial stents implanted in the parent artery is a porous tubular mesh made of nitinol or other alloys [3]. The Enterprise, LVIS and Pipeline are three commercial stents to perform endovascular treatment, the LVIS stent provides a higher degree of metal coverage (approximately 23 %) which is more dense than the conventional Enterprise (8 %) but slightly lower than the Pipeline (approximately 30–35 %). To begin with, the “normal stent” such as Enterprise and LVIS refers to the stent with high porosity whose main function was avoiding coil herniation. Furthermore, the flow diverter (FD) as a special kind of stent is characterized with low porosity which has obvious hemodynamic effect on aneurysms, the pipeline device (PED) was also one of the typical flow diverters (FDs) in our research [4, 5].

The introduction and evolution of various stent systems has greatly broadened the applicability of endovascular therapy [6–9]. The Low-profile Visualized Intraluminal Support device (LVIS®D; MicroVention-Terumo, Tustin, CA, USA) is a novel, self-expandable braided stent with closed cell construction of nitinol material. The metal coverage(surface area covered by the stent) of the LVIS stent is significantly higher than the conventional Enterprise® (Cordis Neurovascular, Miami, Florida, USA) but slightly lower than the Pipeline (Coviden/ev3 Neurovascular, Irvine, CA, USA). Although this stent has been proved to be a safe and effective support device for stent-assisted coil embolization [10–12], there are no studies that evaluate the flow diversion effects of the LVIS stent. In previous studies, computational fluid dynamics (CFD) studies focused on flow diverting effects by comparing normal stent and FD [5, 13, 14]. However, it is necessary to evaluate the difference of hemodynamic effect among the novel LVIS stent, the conventional Enterprise stent and the PED as they are commonly used in the clinical practice. Moreover, in previous studies, the low porosity of flow diverters can be achieved by overlapping stents [4, 15]. Turner et al. reported three cases treated with LVIS stents and they deployed two LVIS stents to reconstruct the fusiform supraclinoid ICA in the case 2 [16]. However, it was still unknown whether the overlapping LVIS stents could provide a similar hemodynamic effect to the PED. Therefore, we present quantitative data by comparing three kinds of commercially available stents: Enterprise, LVIS, and Pipeline in three patient–specific models. Moreover, we verify the differences of flow reduction effects between multiple LVIS stents and FDs.

## Methods

### Aneurysm

We simulated three aneurysms constructed from 3D digital subtraction angiography (DSA). The aneurysms were stratified across the established size categories based on the International Subarachnoid Aneurysm Trial (ISAT), including small (≤10 mm), large (15–25 mm), and giant (≥25 mm). The size was measured on DSA. Case 1 involved a small aneurysm with a longitudinal diameter of 5.12 mm and a neck width of 5.08 mm. Case 2 involved a large aneurysm with a longitudinal diameter of 18.78 mm and a neck width of 11.18 mm. Case 3 involved a giant aneurysm with a longitudinal diameter of 25.16 mm and a neck width of 12.59 mm. The average diameter of the parent vessel is 3.76 mm (Case 1), 4.03 mm (Case 2), 4.19 mm (Case 3) respectively. The medical data were gathered for diagnostic purposes, and the study was approved by the Ethics Committee of our institution.

### Stent modeling and deployment

**Fig. 1:**
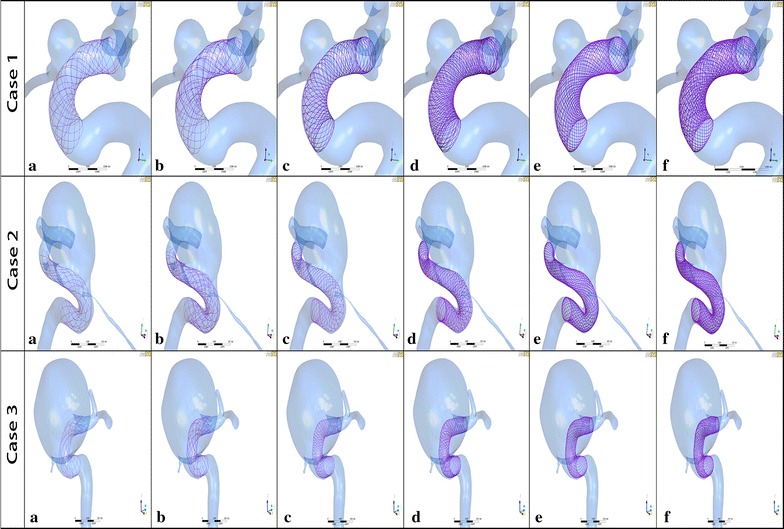
Geometries of aneurysm models of 3 cases after stent placement. **a** A single-Enterprise stent model; **b** double-Enterprise stent model; **c** a single-LVIS stent model; **d** double-LVIS stent model; **e** a single-Pipeline device model; **f** double-Pipeline device model

In our simulations, we developed a novel virtual stenting workflow [17, 18] to deploy the Enterprise, LVIS and Pipeline stents (Fig. 1).

**Fig. 2:**
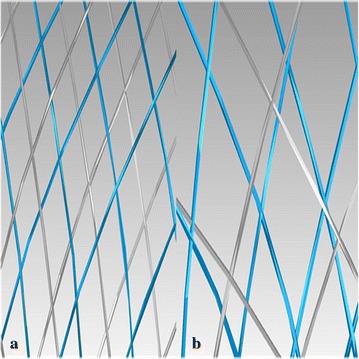
The enlarged stent cells of overlapping stents. The enlarged *blue wires* of the first deployed stent and the enlarged *grey wires* of the second deployed stent were shown in the Geomagic Studio software. **a** The overlapping stents were staggered well; **b** the overlapping stents were not well staggered (disordered or overlapped)

Briefly, the workflow consists of three steps: (1) Pre-processing, which isolates the parent vessel and generates a simplex mesh structure of the maximum inscribed sphere diameter along the vessel centerline using vessel-specific initialization; the simplex mesh is a generalized deformable mesh structure that can expand depending on the relative position of mesh points by applying artificial mathematical forces [19]. It differs from previous approaches by using a non-parametric representation of surfaces and by the addition of deformable contours lying on the surface model. It is semi-automatic, easy to implement and drastically reduces the computational cost of virtual stenting [20] (2) Simplex mesh expansion, where the simplex mesh undergoes radial expansion using mathematical forces, and the deployment stops when the simplex mesh has good apposition with the parent vessel wall; (3) Post-processing that maps the stent pattern on the deployed simplex mesh and sweeps the wires into 3D structures. We chose appropriate overlapping stents that were staggered well (Fig. 2a) by enlarging the 3D cells of stents in Geomagic Studio (version 12.0, Geomagic, Research Triangle Park, NC, USA), while the overlapping stents that were disordered or not well staggered were aborted (Fig. 2b).

### CFD modeling

Computational fluid dynamics modeling was performed by numerically solving the continuity and Navier–Stokes momentum equations for an unsteady blood flow using the commercial software ANSYS CFX 14.0 (ANSYS, Inc., Canonsburg, PA), based on the finite volume method. Fluid volumetric mesh was created and defined by ANSYS ICEM for our simulations. For this calculation, mesh dependency tests were performed to ensure the stability of the simulations; the final grids contained approximately 2 million to 50 million tetrahedral elements for the untreated and stented models. Blood was assumed as an incompressible Newtonian fluid with a density of 1060 kg/m^3^ and a viscosity of 0.004 kg/m/s. Because patient-specific information was not available in the simulations, pulsatile boundary conditions were based on superposition of blood-flow waveforms of the common internal carotid artery using Doppler ultrasonography in normal human subjects for transient analysis. Vessel walls were assumed to be rigid, and no-slip boundary conditions were applied at the lumens. The pressure distribution along the parent artery and in the aneurysm was then computed using the decreases in pressure calculated during the CFD simulations with respect to the P = 10,000 Pa value prescribed at the outlet [21]. Physiologic flow waveforms measured by transcranial doppler were pulsatile. Therefore, the flow data such as (velocity, flow rate and OSS) will change in the whole cardiac cycle. But the patient-specific flow data were not always available in clinical practice, we have to use the representative population average value (1.5 Pa) [22, 23] to eliminate the bias resulted from individual difference. The flow waveforms were scaled to achieve a mean inlet Wall Shear Stress (WSS) over the entire cycle of 1.5 Pa under pulsatile conditions. The unsteady flow solutions were advanced in time using 0.001 s for two cycles with a fully implicit scheme and efficient solution algorithms [24]. Results of the second cycle were used for hemodynamic aneurysm characterization, (e.g., the WSS). Wall shear stress is a tangential drag force per unit area of endothelial surface Aneurysm pressure and the velocity of the perpendicular plane (perpendicular to the aneurysm inlet/neck) of the aneurysm corresponding to the pre-and post-implantation models were calculated and compared at the systolic peak.

## Results

**Fig. 3:**
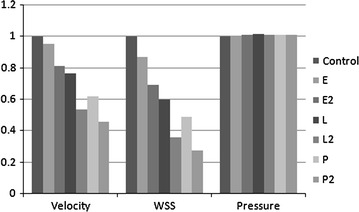
The percentage of hemodynamic changes for stent models. *E*, a single-Enterprise stent model; *E2*, double-Enterprise stent model; *L*, a single-LVIS stent model; *L2*, double-LVIS stent model; *P*, a single-Pipeline device model. Note: *WSS* wall shear stress; Histogram respectively shows the hemodynamic parameter (Velocity, WSS and Pressure) changes among *E*, *E2*, *L*, *L2*, *P*, *P2* compared to Control from *left* to *right*. Although the double Pipeline device (PED) demonstrates the most reduction of Velocity and WSS, the double LVIS stent still demonstrate an obvious flow diversion effect than the conventional double Enterprise stent and the single PED. The overall Pressure change is minor after stenting compared to Control

We first made an average of hemodynamic parameters of three cases and then construct a histogram, i.e., Fig. 3, to demonstrate the percentage of hemodynamic changes (Velocity, WSS and Pressure) after six stent models: single Enterprise, double Enterprise, single LVIS, double LVIS, single Pipeline and double Pipeline compared to Control from left to right. It shows quantitative results in velocity and WSS of the six stent model compared with Control. The hemodynamic values for the Control were shown in Table 1. Significant reduction in WSS (63.88 %, 1.2 Pa) and velocity (46.05 %, 0.0612 m/s) was observed in the double-LVIS stents. A single Pipeline showed less reduction in WSS (51.08 %, 0.96 Pa) and velocity (37.87 %, 0.0503 m/s) compared with double-LVIS stents. The double-Pipeline stents resulted in the most reduction in WSS (72.37 %, 1.36 Pa) and velocity (54.26 %, 0.0721 m/s). A single LVIS stent caused more WSS reduction than double Enterprise stents (39.96 %, 0.75 Pa, *vs.* 30.51 %, 0.57 Pa) and velocity (23.13 %, 0.0307 m/s *vs.* 18.64 %, 0.0248 m/s). Moreover, the pressure increased after stenting, with minuscule extent compared with the unstented model.

**Table 1:** The hemodynamic values for the control

Control	Velocity (m/s)	WSS (Pa)	Pressure (Pa)
Case1	0.0796	0.6989	12,212.1
Case2	0.0879	1.1183	11,158.1
Case3	0.2312	3.8205	10,448.2
Mean	0.1329	2.4694	11,272.8

### Case 1

**Fig. 4:**
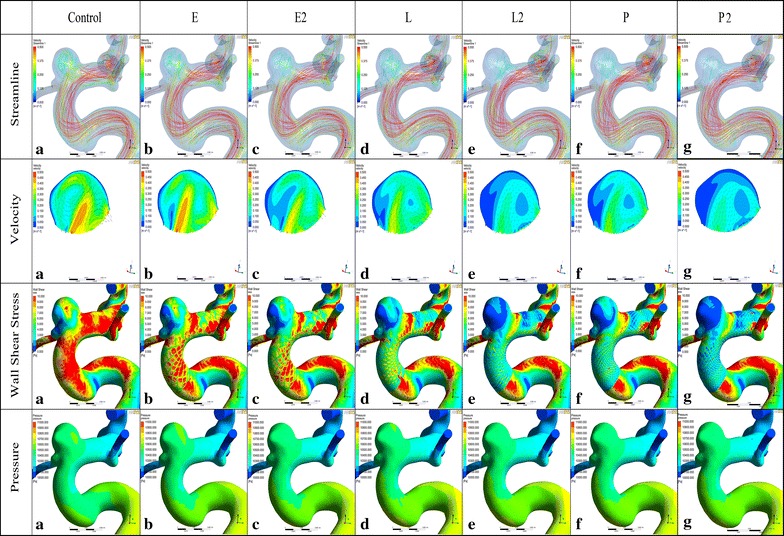
Visualized results of numerical simulation (Streamline, Velocity, WSS and Pressure) of Case 1. *E*, a single-Enterprise stent model; *E2*, double-Enterprise stent model; *L*, a single-LVIS stent model; *L2*, double-LVIS stent model; *P*, a single- Pipeline device model. *E* caused only a small change in the overall intra-aneurysm flow pattern compared with Control. *L* produced a more obvious flow reduction than *E2*. With additional LVIS stent deployment, further reduction was achieved which was more than that of *P*. While *P2* showed the most evident WSS and flow velocity reduction compared with other single and double stent models. The vortex shape seen in Control persists with the gradually lower intensity after stenting similar to the WSS and velocity changes. The WSS of the parent vessel decreased as one goes from *E* to *L2*. While the *L2* showed a bigger WSS decrease than *P*. Note: *WSS* wall shear stress

With no stent, the complex blood flow entered into the aneurysm dome from the mid to proximal neck area in a counterclockwise direction, and a single-vortex flow pattern was observed in the aneurysm dome. After stenting, the complex flow pattern was dampened with no change of vortex pattern but with the gradually lower intensity (Fig. 4). A single Enterprise stent (E) caused only a small change in the overall intra-aneurysm flow pattern. The WSS values changed from 3.8205 to 3.3316 Pa, a 12.79 % reduction. The flow velocity changed from 0.2312 to 0.2231 m/s, a 3.49 % reduction. Although double Enterprise stents (E2) showed a relatively obvious decrease of 31.19 % (1.1916 Pa) and 22.07 % (0.0510 m/s) in WSS and flow velocity, a single LVIS model (L) produced a more obvious flow reduction of 40.3 % (1.5397 Pa) and 26.53 % (0.0613 m/s), respectively. With additional LVIS stent deployment, further reduction in WSS (68.16 %, 2.6042 Pa) and velocity (53.35 %, 0.1233 m/s) was achieved which was more than that of a single Pipeline model (P) (WSS 51.07 %, 1.9510 Pa; velocity 44.81 %, 0.1104 m/s). Meanwhile, double Pipeline devices (P2) showed the most evident WSS and flow velocity reduction of 74.49 % (2.8460 Pa) and 60.15 % (0.1391 m/s) compared with other single and double stent models.

### Case 2

**Fig. 5:**
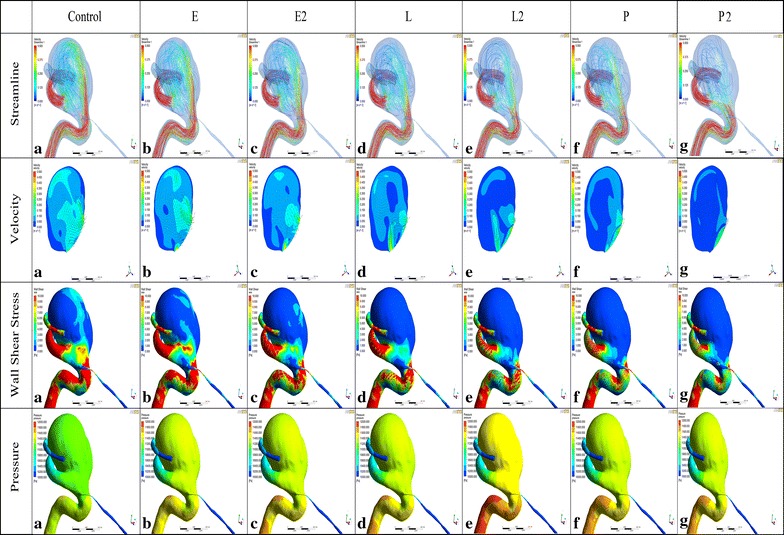
Visualized results of numerical simulation (Streamline, Velocity, WSS and Pressure) of Case 2. *E*, a single-Enterprise stent model; *E2*, double-Enterprise stent model; *L*, a single-LVIS stent model; *L2*, double-LVIS stent model; *P*, a single-Pipeline device model. The number of streamlines, WSS and velocity decreases in the following order: *E* < *E2* < *L*<*P* < *L2* < *P2*. The vortex shape seen in Control persists with the gradually lower intensity after stenting similar to the WSS and velocity changes. The high WSS region near the aneurysmal neck decreased and low WSS region newly generated at the parent vessel after stenting. Compared to Control, two new vortices were formed in L while only one vortex remained in *L2*. Note: *WSS* wall shear stress

From the velocity vectors plotted on the perpendicular plane of each aneurysm model to visualize aneurysm flow patterns, two distinct vortices were observed in the unstented aneurysm. Another two vortices were formed at the dome and the lower left of the perpendicular plane when a single Enterprise (E) or LVIS (L) stent was deployed. However, the vortex at the lower right of the perpendicular plane was dampened when stented with a double Enterprise stent and only one vortex remained when implanted with double LVIS (L2) stents. With a single Pipeline model (P) or double Pipeline devices (P2) deployed, the vortex at the lower right of the perpendicular plane was dampened and another vortex formed at the lower left of the perpendicular plane. As shown in Fig. 5, the number of streamlines became smaller in the following order: a single Enterprise stent (E), double Enterprise stents (E2), a single LVIS stent (L), a single Pipeline device (P), and double LVIS stents (L2) and double Pipeline devices (P2). With a single Enterprise stent, the WSS value changed from 1.1183 Pa to 0.9808 Pa, a 12.30 % reduction. The velocity changed from 0.0879 m/s to 0.0839 m/s, a 4.64 % reduction. Further WSS and velocity reduction was obtained when double Enterprise stents (E2) were deployed (31.60 %, 0.3534 Pa and 18.32 %, 0.0161 m/s; respectively). However, the WSS value and velocity were decreased by 44.50 % (0.4976 Pa) and 25.92 % (0.0227 m/s) when a single LVIS stent (L) was implanted. Moreover, although a single Pipeline device (P) resulted in a WSS and velocity reduction of 55.03 % (0.6154 Pa) and 37.83 % (0.0332 m/s), the reduction became 57.69 % (0.6451 Pa) and 44.88 % (0.03394 m/s) with double LVIS stents (L2). With the double Pipeline devices implanted, the most reduction in WSS (70.95 %, 0.7935 Pa) and velocity (52.74 %, 0.0464 m/s) was obtained.

### Case 3

**Fig. 6:**
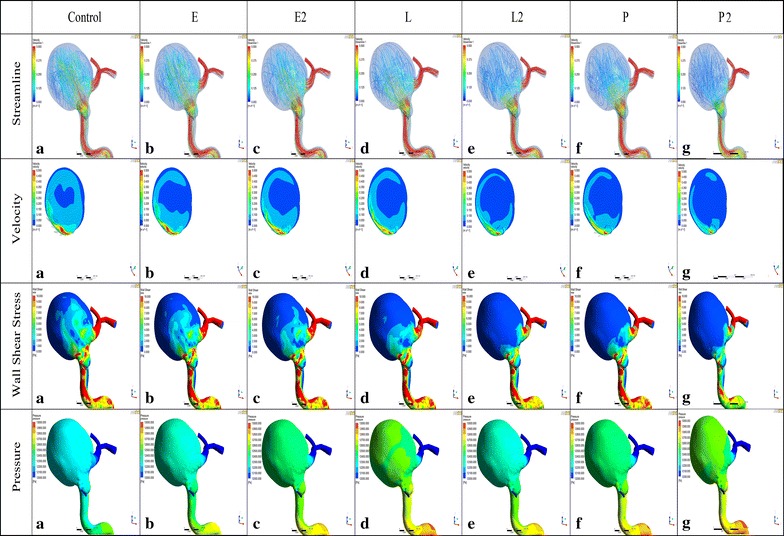
Visualized results of numerical simulation (Streamline, Velocity, WSS and Pressure) of Case 3. *E*, a single-Enterprise stent model; *E2*, double-Enterprise stent model; *L*, a single-LVIS stent model; *L2*, double-LVIS stent model; *P*, a single-Pipeline device model. *P2* was really better at reducing velocity and WSS than *L2*. Nevertheless, *L2* still demonstrate an obvious flow diversion effect than the conventional *E* and *P*. The *vortex shape* seen in Control persists with the gradually lower intensity after stenting similar to the WSS and velocity changes. The low WSS region of the parent vessel increased compared to the Control. A single vortex was observed in the Control model and the vortex showed minimal changes after a single stent or *E2* placement. After *L2* and *P2* stenting, no obvious vortex was observed ultimately. Note: *WSS* wall shear stress

The velocity vector and contour for each model is summarized in the second line of Fig. 6. A single vortex was observed at the lower left of the perpendicular plane of the control model and the vortex showed no or minimal changes after a single stent or a double Enterprise stent (E2) placement. After the double LVIS stent (L2) and the double Pipeline devices (P2) stenting, no obvious vortex was observed ultimately. A single Enterprise stent (E) reduced the WSS from 0.6989 to 0.6083 Pa, a 12.98 % reduction; and the velocity from 0.0796 to 0.0732 m/s, a 7.96 % reduction. These were further reduced by a double Enterprise stent (25.09 and 8.95 %, respectively). However, the single Pipeline device (P) resulted in further reduction of WSS and velocity (30.83 %, 0.3136 Pa and 10.23 %, 0.0142 m/s) than a double Enterprise stent (E2). Moreover, the double LVIS stent (L2) showed the greatest reduction of WSS and velocity (50.34 %, 0.3518 Pa and 26.20 %, 0.0209 m/s), while a single Pipeline device (P) led to a 44.87 % (0.3136 Pa) and 17.79 % (0.0142 m/s) decrease, respectively. The greatest reduction of WSS (62.98 % 0.4402 Pa) and velocity (338.84 % 0.0309 m/s)was observed when the double Pipeline devices (P2) implanted.

## Discussion

The LVIS stent, regarded as a novel self-expandable stent of smaller cell size (~0.9 mm) than currently available coil-assist stents [11, 16], has been used successfully to treat wide-necked and dissecting aneurysms [25, 26]. It may provide better protection against coil protrusion and yields improved flow diversion. Furthermore, the LVIS stent is well-visualized throughout its course, owing to dual radiopaque helical strands [26]. The Food and Drug Administration (FDA) and the management of our country set the indication of Pipeline device (PED) to treat large or giant wide-necked intracranial aneurysms in the internal carotid artery from the petrous to the superior hypophyseal segments [27]. However, in addition to the indication of PED, the novel LVIS stent also offer promise in treating more types and locations (especially for the posterior circulation) of aneurysms compared with the PED [28, 29]. The LVIS stent is denser than the conventional Enterprise stent, the porosity of the stent is of key importance for hemodynamic modifications as it controls resistance to blood flow through the interwire gaps, thereby allowing the possibility of flow stasis in the aneurysm; hence promoting rapid thrombosis [30–32] Moreover, the intra-aneurysm thrombosis after embolization could be hampered by high-speed flow and high WSS while the stent could decrease the flow velocity and the WSS [18, 33, 34]. So it is necessary to evaluate the difference of hemodynamic effect between the LVIS stent and the Enterprise stent. Therefore, our purpose was to measure the effects of the LVIS stent on aneurysm hemodynamics and to quantify the difference when the LVIS stent was compared with the Pipeline device and the Enterprise stent. It would be helpful to enhance current understanding of these stents and how they can best be used to treat cerebral aneurysms which would provide some reference information for physicians.

Currently, there is a lot of research interest on hemodynamic changes associated with flow diverters and multiple conventional stents. Tremmel et al. [13] simulated the various combinations of one to three conventional Enterprise or Vision stents and demonstrated a 14.1 and 28.7 % WSS reduction with a single Enterprise and double Enterprise stent, which is similar to our study. The WSS reduction of a single Enterprise and double-Enterprise were 12.72 and 30.51 % respectively. Levitt et al. [14] demonstrated that aneurysm treatment with a Pipeline device reduced blood flow and hemodynamic shear stress in the aneurysm dome and that the WSS at the moment of peak systole was reduced by 51.89 %, similar to our results (51.08 %). Kojima et al. [5] virtually modeled three kinds of commercially available intracranial stents (Enterprise, Silk and Pipeline) in an intracranial ophthalmic artery aneurysm. Consistent with our results, they also showed that the reduced velocity within the aneurysm sac with a double Enterprise stent is not as significant as the flow diverters. However, no study has quantified the effect of the LVIS stent on aneurysm hemodynamics, although the LVIS stent has generated good clinical improvement with high levels of occlusion and low rates of recanalization [10]. In our study, the single LVIS stent caused more flow reductions than the double Enterprise stent, but less than the Pipeline device. Nevertheless, the double LVIS stent resulted in a better flow diverting effect than a Pipeline device. Finally, we found that the LVIS stent has certain hemodynamic effects (obvious reductions in WSS and velocity) on cerebral aneurysms compared with the Enterprise and Pipeline stents.

In the practical daily clinical setting, it is very difficult to quantify the hemodynamic effects of stents, because of multiple uncertainties and fluctuating factors. Furthermore, a real case can only be treated with only one option in clinical practice, so we would have been unable to simulate all stents or flow diverters in identical cases. However, in virtual models, we can use the virtual stenting technology to compare the options before the interventionist actually performs the procedure. In our study, we virtually modeled three kinds of commercially available intracranial stents (LVIS, Pipeline and Enterprise). Kojima et al. demonstrated that the mesh characteristics, like size and pore density affected the blood flow in the aneurysm [5]. In other words, the reduction effect of velocity and wall shear stress is in proportion to the stent’s metal coverage. The biological basis for the reduction may be that the stent mesh could block the blood into aneurysm and break intra-aneurysmal blood circulation, which leads to decrease of intra-aneurysmal velocity and thereby allowing the possibility of flow stasis in the aneurysm, hence increasing blood viscosity and promoting rapid thrombosis [30]. Then neointima gradually formed over the stent surface to completely exclude the aneurysm from the circulation [35]. Ultimately, the aneurysm was cured. However, Xiang et al. found a large hemodynamic difference between two adjacent aneurysms in case 3 after a single pipeline implantation despite identical metal coverage [36]. Therefore, although the metal coverage was known, it was also necessary to quantify the hemodynamic factors.

In our study, we verified the differences of flow reduction effect for aneurysms after stent placement and the results showed that a single LVIS placement was better than a double Enterprise stent in reducing the velocity and WSS of the aneurysm sac. We also found that a double-LVIS stent was better than a single Pipeline device and that there was a pressure increase after stenting but this change was minuscule for all the stents. The decreased flow velocity could be indicative of stagnant blood flow, which can promote thrombosis. Wall shear stress has been found to play an important role in the recurrence of aneurysms [37, 38]. Areas with low velocity and WSS are subject to increased chance of thrombus [20, 24]. High-flow conditions may significantly contribute to aneurysm recanalization via multiple mechanisms. To begin with, intra-aneurysm thrombosis after embolization could be hampered by high-speed flow and high WSS [33, 34]. Furthermore, high blood flow at the treated aneurysm neck may delay neointima formation over the stent surface and lead to coil compaction seen at follow-up [39, 40]. In the previous studies, many researchers [18, 36, 41] have focused on the flow diversion properties after stenting to evaluate the recurrence risk and found that an emerging or residual local high WSS or velocity were prone to future recanalization. However, these studies based on a small sample could not provide accurate gold standard values to predict the late recurrence and we only performed a relevance research to evaluate hemodynamic changes after stenting. Further study based on a large sample should devote more attention to identify the gold standard values. The main finding of our study was a decreased velocity and WSS after stent placement. We also quantified the differences of flow reduction effect for aneurysms after stent placement and the results, indicating a reduced risk of recurrence. It would be beneficial to choose appropriate therapeutic schedule in clinical practice. Meanwhile, sub-3 % pressure increase after stenting may be due to several reasons: when the stent was implanted, the flow resistance increased, the outlet pressure in our computational models was set to be a constant 10,000 Pa, the pressure at the inlet and the aneurysm increased in order to keep the flow rate steady; moreover, according to the Bernoulli’s Principle, the reduced velocity in the aneurysm would increase pressure after stenting; the increment of pressure inside the aneurysm was small compared to 10,000 Pa, thus the pressure change after stenting was minuscule.

This study has several limitations. First, the flow simulation of three patient-specific aneurysms with different stent configuration and the hemodynamic parameters in peak systole may not be sufficient to demonstrate generalized results. It is necessary to gather more data on large numbers of aneurysms of various sizes and morphologies. Second, the mechanisms of aneurysm occlusion cannot be explained simply by hemodynamics, other factors such as biological factors (blood residence time) in pathophysiology are also indispensable. Third, the resulting pattern of overlapping wires and cells would vary if one stent was deployed inside the other, which makes overlapping stents unpredictable. Last, several assumptions, such as rigid wall, laminar flow, Newtonian blood, and constant pressure at outlets may affect the hemodynamic results.

## Conclusions

This is the first study analyzing flow modifications associated with placement of LVIS stents. In our study, a single LVIS stent caused more flow reductions than the double-Enterprise stent but less than a Pipeline device. Nevertheless, the double-LVIS stent resulted in a better flow diverting effect than a Pipeline device.

## Abbreviations

CFD: computational fluid dynamics
WSS: wall shear stress
LVIS: Low-Profile Visualized Intraluminal Support
PED: the pipeline device
FD: flow diverter
